# Multi-Stage Temporal Convolution Network for COVID-19 Variant Classification

**DOI:** 10.3390/diagnostics12112736

**Published:** 2022-11-09

**Authors:** Waseem Ullah, Amin Ullah, Khalid Mahmood Malik, Abdul Khader Jilani Saudagar, Muhammad Badruddin Khan, Mozaherul Hoque Abul Hasanat, Abdullah AlTameem, Mohammed AlKhathami

**Affiliations:** 1 Department of Software, Sejong University, Seoul 05006, Korea; 2 CORIS Institute, Oregon State University, Corvallis, OR 97331, USA; 3 Department of Computer Science and Engineering, Oakland University, Rochester, MI 48309, USA; 4 Information Systems Department, College of Computer and Information Sciences, Imam Mohammad Ibn Saud Islamic University (IMSIU), Riyadh 11564, Saudi Arabia

**Keywords:** artificial intelligence, COVID-19, deep learning, genomes sequence analysis, variant classification

## Abstract

The outbreak of the novel coronavirus disease COVID-19 (SARS-CoV-2) has developed into a global epidemic. Due to the pathogenic virus’s high transmission rate, accurate identification and early prediction are required for subsequent therapy. Moreover, the virus’s polymorphic nature allows it to evolve and adapt to various environments, making prediction difficult. However, other diseases, such as dengue, MERS-CoV, Ebola, SARS-CoV-1, and influenza, necessitate the employment of a predictor based on their genomic information. To alleviate the situation, we propose a deep learning-based mechanism for the classification of various SARS-CoV-2 virus variants, including the most recent, Omicron. Our model uses a neural network with a temporal convolution neural network to accurately identify different variants of COVID-19. The proposed model first encodes the sequences in the numerical descriptor, and then the convolution operation is applied for discriminative feature extraction from the encoded sequences. The sequential relations between the features are collected using a temporal convolution network to classify COVID-19 variants accurately. We collected recent data from the NCBI, on which the proposed method outperforms various baselines with a high margin.

## 1. Introduction

Millions of people have been affected by the SARS-CoV-2 virus worldwide due to its outbreak in Wuhan, China, and its spread worldwide [1]. When a new virus emerges, it is essential to determine its cause in order to address pandemics rapidly. This encourages researchers to categorize new viruses, such as SARS-CoV-2, correctly, and further discover the causes of their spread. To detect and limit the spread of viruses and their variants, suitable procedures and controls must be developed, and this categorization is crucial in doing so. SARS-CoV-2 identification is challenging due to its genetic similarities with another Coronaviridae virus family, yielding a high ratio of false positives in detection [2]. Metsky et al. [3] state that patients with suspected SARS-CoV-2 have symptoms of other respiratory viral infections. Domain experts are therefore motivated to correctly differentiate between SARS-CoV-2 and other related viruses in order to improve the patient diagnostic process and manage the spread of the virus in the future. There is a single-stranded RNA virus called SARS-CoV-2, which is encapsulated, positive-sense, and has a genome of around 30 kilobases [4]. Generally, RNA viruses have a relatively high mutation rate [5]. Genetic mutation can rarely happen among viruses of identical species in diverse lineages. As a result, mutated viruses may occasionally trigger an infection outbreak in a population, as in SARS-CoV-2. The symptoms of coronavirus infection can include fever, breathing difficulties, and pneumonia, which are the most common symptoms of the disease, caused by zoonotic transmission to humans [6]. SARS-CoV-2’s transmission from person to person has also been confirmed, and its genetic characteristics have recently been identified by utilizing next-generation sequencing and metagenomic analysis [7,8].

The whole genome of SARS-CoV-2 has been studied, and the results of this analysis have led to the conclusion that SARS-CoV-2 is most closely associated with two bat-borne coronaviruses with functions similar to SARS. This conclusion has been supported by several studies performed based on viral proteins [1,2]. SARS-CoV-2 shares significant similarity with the bat coronavirus RaTG13, according to a phylogenetic study of whole genome alignment and a similarity plot [3]. Furthermore, another study [4] recently discovered that SARS-CoV-2’s receptor-binding domain (RBD), similar to other Sarbecovirus strains, is able to interact with the host receptor angiotensin-converting enzyme 2 (ACE2) exactly as other Sarbecovirus strains do, which lends credence to the idea that the virus was originally isolated from bats [5,6]. In line with its identical genetic structure to other viruses in the same family, SARS-CoV-2 exhibits similar symptoms, and it is difficult to predict the disease early on because of its similar symptoms to other viruses in the same family. Ozturk et al. [7] utilized a deep neural network to automatically identify cases of SARS-CoV-2 by using X-ray images as input to the neural network. As a result of the preliminary findings, it was discovered that the approach is capable of predicting COVID-19 and no findings with 98.08 percent accuracy and 87.02 percent accuracy in the prediction of various classes (COVID-19 and no findings combined with pneumonia). The results of another study [8] demonstrate that deep learning techniques can identify age-related macular degeneration (AMD), a leading cause of blindness in the elderly. According to the study’s findings, the average area under the curve (AUC) value is 0.85. However, in previous studies, authors [9] utilized deep learning mechanisms to classify better whether diseases are associated with mutations in the DNA-binding domains of proteins. In the mentioned study, the prediction accuracy is 0.82, and the AUC is 0.90. This shows that deep learning can offer predictions at high levels of accuracy, which is compelling evidence for its potential. The results of these studies led us to design a deep-learning-based prediction approach to identify the pathogenic genetic sequences of viral strains. The main contributions of this study can be summarized as follows:

- Currently, many methods for classifying miRNAs rely on manual feature extraction to be successful. The two main types of methods used for analyzing pre-miRNAs, either focusing on their spatial or sequential structure, are ineffective. We propose a temporal convolution neural network to learn spatiotemporal relationships and accurately identify different variants of COVID-19.
- A sequence is represented by both labels and encoding, which keep track of nucleotide positions within the sequence. We convert this information into a numerical description of the nucleotide position within the sequence.
- We conduct a detailed ablation study on both deep learning architectures as well as machine learning algorithms for classifying DNA sequences based on a deep learning architecture.
- The proposed framework is validated on challenging COVID-19 sequences and achieves state-of-the-art results for classification.

The remainder of the discussion in this article is organized as follows. In Section 2, the literature review is briefly summarized. We explore the suggested model in greater depth in Section 3. Section 4 presents the experiment’s findings and an analysis of the findings. The conclusion of this research is provided in Section 5.

## 2. Literature Review

Genome sequence classification methods have traditionally been based on alignment-based techniques, such as the Basic Local Alignment Search Tool (BLAST), which exploits similarity by searching for local alignments [10], and the Burrows–Wheeler Aligner (BWA) [11]. The primary purpose of these techniques is to annotate the viral genes in order to detect them [12]. Several successful alignment-based methods, such as BLAST, have been used to identify sequence similarity [13]. Nonetheless, when these methods are applied to thousands of complete genomes to analyze a given genome, they require a long computation time, and it is not practical to use them in real life [14]. According to the authors of both studies [13,15], it is suggested that the alignment of the genes should be conducted assuming that they are homologous, i.e., that their continuous structure is similar. Nevertheless, this is often not the case when it comes to real situations. Deoxyribonucleic acid (DNA) binding to proteins is predicted using several alignment-free computational approaches [16,17]. There is no requirement for gene alignment in order to predict and model proteins with DeepFam [18]. Compared to methods that use sequence alignments to predict binding proteins, DeepFam uses a feedforward convolutional neural network. DeepFam is found to have better accuracy and requires a shorter time for execution than methods that use the alignment of sequences [18]. Similarly, Machine Learning with Digital Signal Processing Graphical User Interface (MLDSP-GUI), developed by Randhawa et al. [15], can also be used to compare and analyze DNA sequences without requiring alignment. According to the authors, this tool is developed to address alignment issues associated with DNA sequences. In another study, Ullah et al. introduced a framework for accurately classifying splice site predictions. In this model, the authors extract useful patterns from DNA sequences, convert them into numerical descriptors, and pass them to various machine learning algorithms. Zhang et al. [19] investigated various deep-learning-based techniques, such as N-gram probabilistic, DNN, and the CNN model, to identify DNA sequences, and a novel technique to extract features from random DNA sequences is proposed to measure the distance among the nucleotides. Lastly, they evaluated their paradigm on four different viral genomic datasets: hepatitis C, AIDS, influenza, and COVID-19 [19]. In another study, the extreme gradient boosting algorithm was used to classify mutated DNA sequences in order to recognize the derivation of viruses. This method achieved an accuracy rate of 89.51 percent when classifying DNA sequences by employing a hybrid technique involving XGBoost learning [20]. The area under the curve for predicting N4-methylcytosine based on DNA sequences reached a significant value greater than 0.9 using the feature selection and stacking technique of a deep learning model [21]. The study in [22] investigated linear classifiers such as logistic regression, linear SVM, multinomial Bayes, Markov to identify the limited and whole genomic sequences of the HCV dataset. The authors tested and assessed the findings of a variety of K-mer sizes [22]. Rincon et al. [23] presented a technique for predicting SARS-CoV-2 with 100 percent specificity using a deep learning architecture. Various detection methods have been described in the literature for detecting COVID-19 based on DNA sequences, such as microarray, polymerase chain reaction, and isothermal-based methods based on the combination of these methods. However, these methods require a great deal of time and money because they must be performed in a laboratory environment. Several factors distinguish the present study from other studies, but the most striking characteristic is the fact that it offers a low-cost machine-learning-based approach to detecting COVID-19 from DNA sequences, without the need for a laboratory setting. Currently, there is no published study demonstrating that DNA nucleotide signals can be used to predict COVID-19 variants. The present research aimed to develop a deep-learning-based computer-aided tool to effectively classify COVID-19 variants. As a result of this study, the authors introduced a novel procedure to improve code understanding, differing from the DNA sequence obtained from the experimental study.

## 3. Proposed Methodology

**Figure 1:**
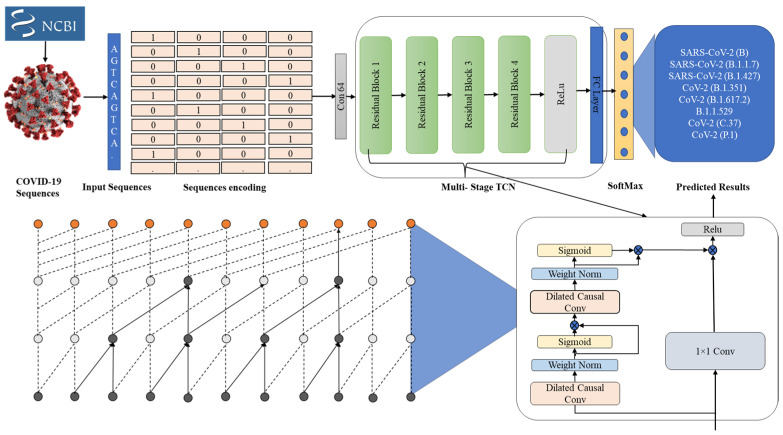
The proposed framework for COVID-19 virus variant classification.

We cover the technical specifications of the proposed framework in this section. In Figure 1, we demonstrate the general flow of our proposed framework, which is divided into three main phases. The first phase involves collecting sequence data from the National Center for Biotechnology Information (NCBI) for COVID-19 virus variants. The preprocessing of these sequences is performed in the second and final phases, which is used to classify these sequences accurately.

### 3.1. Genomic Sequence Data Collection

The National Centre for Biotechnology Information (NCBI) (https://www.ncbi.nlm.nih.gov, accessed on 10 December 2021) is a public database of nucleotide sequences where one may access the whole DNA/genomic sequences of viruses, including MERS, SARS, COVID-19, influenza, hepatitis, and dengue. The DNA sequence data can be downloaded in FASTA format, and the lengths of the sequences range from 8 to 37,971 nucleoids, depending on the type of sequence. 

### 3.2. Genomic Sequence Data Preprocessing

Before applying machine learning and deep learning techniques to raw genomic data, it is essential to preprocess the raw data to allow the technique to work with numerical rather than categorical data types. In the DNA dataset, there is a categorical sequence of genomic information. There are numerous techniques to convert categorical data into numerical data. The process of transforming categorical nucleotide data into numerical form is known as encoding. In this study, the DNA sequence is encoded using both label encoding and genomic sequence encoding. Our investigations revealed that the encoding technique significantly affected the accuracy of the classification process. Label encoding refers to the assignment of an index number to each nucleotide within a DNA sequence (A-1, G-2, C-3, and T-4), which describes the sequence in most detail. The DNA sequences are converted into numbers using Label Binarize.

### 3.3. Temporal Sequential Learning Mechanism for COVID-19 Variants

**Single-stage TCN:** In recent years, deep learning approaches have received more attention in a wide range of domains, such as surveillance systems [24,25] and medical image analysis [26,27]. We use only temporal convolutional layers in our single-stage model. Our model does not employ pooling or fully connected layers, reducing the temporal resolution and significantly increasing parameter numbers. SS-TCN refers to the single-stage temporal convolutional network. TCNs with only one stage have a first layer, a 1 × 1 convolutional layer, which adjusts the dimension of the features in the network to match the number of elements. In the next layer, dilated 1D convolution layers are added. Our architecture is inspired by the wavenet architecture [28], where the dilation factor is doubled for every layer, i.e., 1, 2, 4, …, 512. We use the same number of convolutional filters across all layers. In contrast to wavenet, we apply causal convolutions with a 3D kernel, instead of the causal convolutions used in wavenet. With each layer, the previous layer’s output is convoluted with dilated activation using ReLU. Further, we utilize residual connections to allow gradients to flow smoothly. The following is a formal description of each layer’s operations:

(1) $${ℋ}_{\ell }=ReLU\left(W_{d}*\;{ℋ}_{\ell -1}+b_{d}\right),$$

(2) $${ℋ}_{\ell }={ℋ}_{\ell -1}+W*{ℋ}_{\ell }+b,$$

A dilated convolution filter with a kernel size of 3 is described by $W_{d}\;\epsilon \;{ℛ}^{3\times D\times D}$ as follows: ${ℋ}_{\ell }$ is the output of Layer ℒ, * denotes the convolution operator, D is the value of the kernel size, and $W_{d}\;\epsilon \;{ℛ}^{3\times D\times D}$ represents the weights of the convolution filter. The weights of convolutional filters are $W\;\epsilon \;{ℛ}^{1\times D\times D}$, and the bias vectors are $b_{d}$, $b\;\epsilon {ℛ}^{\;D}$. In this method, the receptive field is increased by the use of dilated convolution on the same number of layers, without increasing the kernel size or size of the layers. The benefit of using only a few layers is that we can achieve very large receptive fields, since the number of layers increases exponentially with the number of layers in the model, which helps to prevent the model from overfitting the data during training. The receptive field at every layer is determined as follows:

(3) $$ReceptiveField\left(\ell \right)=2^{\ell +1}-1,\;$$

When ℒ is the layer number, the formula is only valid for kernels with a size of 3 when ℓ ϵ [1, ℒ] is the kernel size. The output class probabilities are computed using a convolution of 1×1, followed by softmax activation on the output of the last dilated convolution layer.

(4) $${𝓎}_{𝓉}=softMax\left(Wh_{ℒ,𝓉}+b\right),$$

At time 𝓉, ${𝓎}_{𝓉}$ is the class probability, $h_{ℒ,𝓉}$ is the output of the last dilated convolution layer, and *W* ∈ RC × D $W\;\epsilon \;{ℛ}^{C\times D\;}$, and $b\;\epsilon \;{ℛ}^{C\;}$ are the weights and bias for the 1×1 convolution layer.

**Multi-stage TCN:** It has been demonstrated that stacking several predictors sequentially improves performance in many tasks, including human pose estimation [29,30,31] and surveillance systems. A stacked architecture is composed of multiple models stacked sequentially, where each model operates directly on the previous model’s output. In such a structure, predictions from previous stages are incrementally refined. Taking inspiration from such architectures, we propose a multi-stage temporal convolutional network (MSTCN) for COVID-19 variant sequence classification. Every stage of this multi-stage model refines the initial predictions from the previous stage. Aiming to solve the temporal COVID-19 sequence classification problem, we propose a multi-stage convolutional network based on the success of such architectures. The multi-stage model starts with a basic prediction and refines it at each stage. The following are the sequence-by-sequence features needed for the first stage:

(5) $${𝓎}^{{}^{\circ}}=S_{1:T,}$$

(6) $${𝓎}^{S}=ℱ\left({𝓎}^{S-1}\right)$$

In such a multi-stage architecture, ${𝓎}^{𝒮}$ represents the output at S stages and ℱ is the single-stage TCN discussed in Section 3.1. Multi-stage architectures provide a larger context for making class-label predictions.

**Dual dilated Layer:** MS-TCN includes a number of dilated convolution layers that are combined with a dilation factor that augments as the number of layers increases. As a result of this, the higher layers will have a larger receptive field, while the lower layers will still have a very small receptive field. In addition, due to the high dilation factor in MS-TCN, higher layers operate convolutions around distant time steps to achieve higher resolutions. The dual dilated layer (DDL) is designed to overcome this problem. The DDL combines the dilation of two convolutions by varying the dilation factors as an alternative to receiving a single dilated convolution. Initially, the dilation factor of the first convolution is low in the lower layers, and, as the number of layers increases, the dilation factor exponentially increases. On the other hand, the dilation factor in the second convolution is initially large and exponentially decreases as the number of layers increases. At each layer, there are a number of operations, which can be described in more detail as follows:

(7) $${ℋ}_{\ell ,\;\;d_{1}}=W_{d_{1}}*\;{ℋ}_{\ell -1}+b_{\;d_{1},}$$

(8) $${ℋ}_{\ell ,\;\;d_{2}}=W_{d_{2}}*\;{ℋ}_{\ell -1}+b_{\;d_{2},}$$

(9) $${ℋ}_{\ell }=ReLU\left(\left[{ℋ}_{\ell ,\;\;d_{1}},{ℋ}_{\ell -1}+b_{\;d_{2},}\right]\right),$$

(10) $${ℋ}_{\ell }={ℋ}_{\ell -1}+W*{ℋ}_{\ell }+b,$$

**Figure 2:**
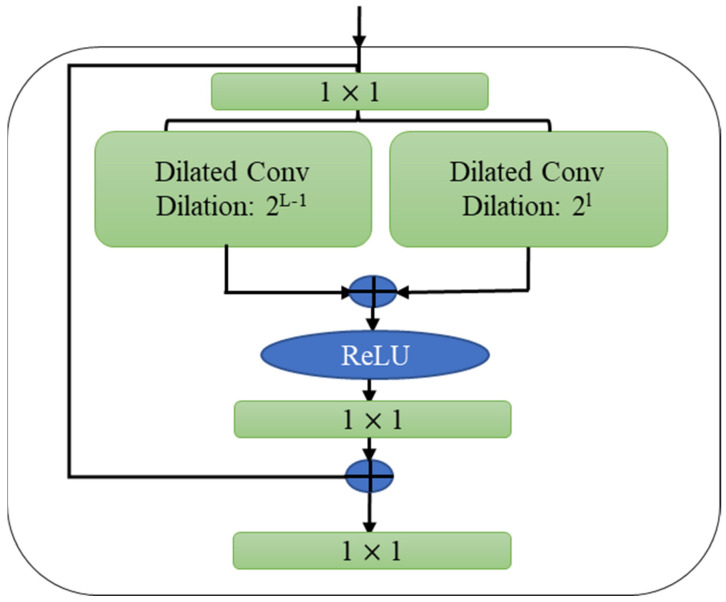
Architecture of dual dilated layers.

A dilated convolution with a dilation factor $2^{\ell }$ and $2^{\ell -1}$ is $W_{d_{1}}$, and $W_{d_{2}}\;\epsilon \;{ℛ}^{3\times D\times D}$, respectively, while a 1×1 convolution has $W\;\epsilon \;{ℛ}^{1\times 2D\times D}$, and the bias vectors are $b_{\;d_{1},}$, $b_{\;d_{2},}$, and $b\;\epsilon {ℛ}^{D}$. As a result of (9), ${ℋ}_{\ell ,\;\;d_{1}}$ and ${ℋ}_{\ell ,\;d_{2}}$ are concatenated. As shown in Figure 2, the dual dilated layer appears above the single dilated layer. In addition to dual dilated layers, feature pyramid networks (FPNs) are also available in the literature for the fusion of multiscale information [32]. They have been successfully applied for temporal action segmentation [33]; the receptive field for these approaches, however, remains very limited, since they have a very narrow focus. Furthermore, an FPN identifies multiscale features through pooling operations, which results in a loss of information necessary to segment temporal data in the FPN outcome. On the other hand, DDL maintains the temporal resolution of the input sequences while simultaneously combining features from various scales.

**MSDDL-TCN:** We describe MSDDL-TCN, which combines the dual dilated layer with the proposed MS-TCN, in this section in an effort to improve MS-TCN. The initial stage of MSDDL-TCN operates in a similar way to MS-TCN in that it generates the preliminary prediction, while the remaining stages refine this prediction incrementally and in turn generate the final prediction. We propose an adaptation of an SS-TCN that employs dual dilated layers in place of the simple dilated residual layers used in SS-TCNs that were initially designed as part of the prediction generation stage. As a result of utilizing this DDL, it is possible to generate better predictions regardless of the layer in which data are collected, as both local and global features can be captured. In order to accomplish refinement more easily than prediction generation, dilated residual layers are incorporated into the SS-TCN architecture. As a result of our experiments, we found that the optimization process works best when DDL is used only during the first stage. There are many advantages to adding more stages to a prediction model, including incremental refinement and a vastly increased number of parameters. In spite of this, since most refinement stages share a common purpose, it may be possible for both parameters to be shared to achieve a more compact model. In our experiments, we found that sharing the parameters between the refinement stages results in a significant reduction in the number of parameters while only slightly degrading the accuracy of the final model. Additional stages can improve predictions incrementally, but they also result in a significant increase in parameters. Since the refinement processes share the same objective, we can use their parameters together to produce a more compact model. As a result of our experiments, it was found that sharing parameters between refinement stages results in a substantial reduction in parameters, leading to only a minor loss of accuracy.

### 3.4. Proposed Model’s Objectives

The proposed model provides a methodology for the correct classification of viral classes using an alignment-free method to calculate the genomic sequences based on the genomic sequences provided for the different pathogenic viruses. For this purpose, the whole genome sequence is first encoded into numerical descriptors, and then these descriptors are passed over a number of convolution layers for the extraction of features from the encoded sequences. In order to classify virus variants in genomic sequences, a vector of features extracted from a multi-stage TCN is passed through.

## 4. Experimental Results

For binary classification models, a model evaluation metric that is commonly used to evaluate the performance of models when data are unbalanced is accuracy. However, the result may be misleading if accuracy is used as the only metric to evaluate the model. The data for classifying COVID-19 genes are unbalanced, because there are 10.3 percent positive samples and 89.7 percent negative samples. Moreover, 96.31 percent of samples are negatively characterized by regulatory motifs for COVID-19 genes in the set of virus genes with regulatory motifs. However, 3.69 percent of samples are positively characterized by regulatory motifs for COVID-19 genes in the set. In these cases, the classifiers may be biased towards the majority class, so the classification may not work well as a result. As a result, the deep learning models are evaluated and compared using a confusion matrix, and the following metrics are calculated.

### 4.1. Method Evaluation

The evaluation of our method was performed on the test dataset in this study. In the analysis, we calculated five different performance metrics: specificity (Spe.), sensitivity (Sen.), accuracy (Acc.) F1 score, and Matthews correlation coefficient (MCC). In order to determine the predictive capability based on the number of true negatives (TN), false negatives (FN), and false positives (FP), the following equations are used:

(11) $$\mathrm{Accuracy}=\frac{𝒯𝒫+𝒯𝒩}{𝒯𝒫+𝒯𝒩+ℱ𝒫+ℱ𝒩}$$

(12) $$\mathrm{Specificity}=\frac{𝒯𝒩}{𝒯𝒩+ℱ𝒫}$$

(13) $$\mathrm{Sensitivity}=\frac{𝒯𝒫}{𝒯𝒫+ℱ𝒩}$$

(14) $$\mathrm{Precision}=\frac{𝒯𝒫}{𝒯𝒫+ℱ𝒫}$$

(15) $$\mathrm{MCC}=\frac{\left(𝒯𝒫*𝒯𝒩-ℱ𝒫*ℱ𝒩\right)}{\sqrt{\left(𝒯𝒫+ℱ𝒫)(𝒯𝒫+ℱ𝒩)(𝒯𝒩+ℱ𝒫)(𝒯𝒩+ℱ𝒩\right)}}$$

### 4.2. Model Implementation

The proposed model is implemented using the Windows 10 operating system installed on an Nvidia RTX-3080 GPU in order to run our experiment. The code was written in Python 3.7 with the deep learning framework Keras 2.3.0, with TensorFlow1.14.0 as the backend. The given DNA sequences were converted into numerical descriptors using a sequence encoding technique. After the sequence encoding in the convolution layer, the first index position is usually taken to denote zero padding, where zero padding is defined for variable-length sequences. We employ a multi-stage model with MSDDL-TCN, a four-stage process with one and three prediction generation and refinement stages, all of them identical, and all composed of the same process. Three dilated convolution layers are included in each of the proposed model stages, and these layers are also present in the refinement stages. In the proposed model, we use a seven-layer structure in order to generate prediction results at the prediction stage. There is a dropout after each layer, with a probability of 0.5, which is used for each layer. There are 128 filters used in each layer of the model, with a filter size of 3. We use the Adam optimizer in all of our experiments, whereby a learning rate of 0.0001 is used.

### 4.3. Dataset

**Figure 3:**
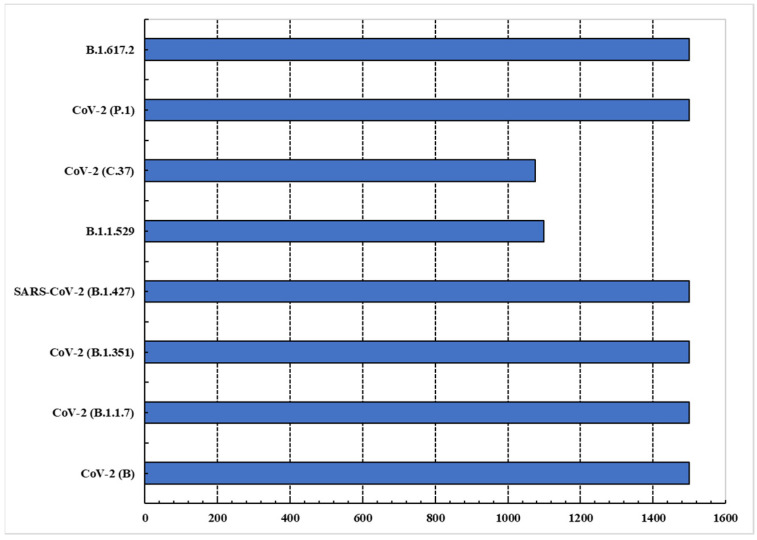
Details of data collected for each COVID-19 variant; *x*-axis shows number of sequences for each variant described on the *y*-axis.

In recent years, the analysis of COVID-19 data has become one of the most important topics, yet the data are not yet available in the form of benchmarks that can be analyzed. For the analysis of COVID-19 data, the existing methods rely on limited virus data from the NCBI website, in addition to other known virus’ data for analysis. However, we collected data from the NCBI regarding our proposed framework that can be evaluated for all COVID-19 variants’ classification. The recent data on each variant were retrieved by searching the NCBI website using the scientific name of the virus variant. In the United States, the Centers for Disease Control and Prevention (CDC), according to their severity rate, have classified these variants into four categories based on their severity levels. These are Variants Being Monitored (VBM), Variants of Interest (VOI), Variants of Concern (VOC), and Variants of High Consequence (VOHC). Our study analyzed data from all categories, as this provided an effective and efficient classification of variants. The statistical details of the proposed dataset are shown in Figure 3.

### 4.4. Results and Discussion

**Figure 4:**
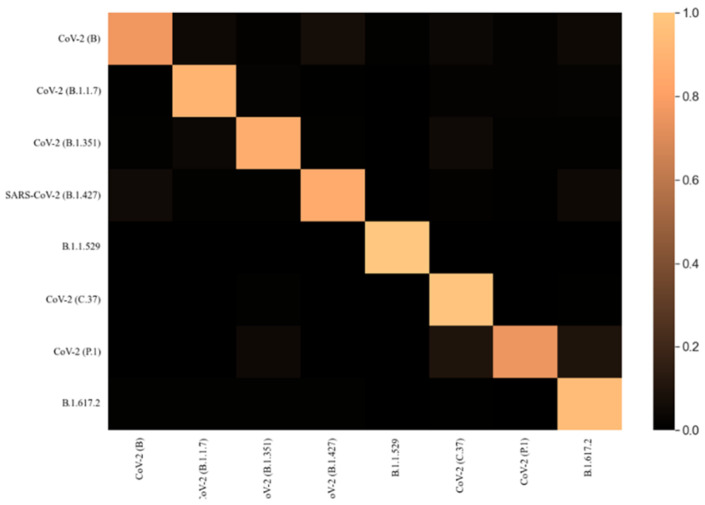
Confusion matrix of the proposed multi-stage TCN framework.

**Figure 5:**
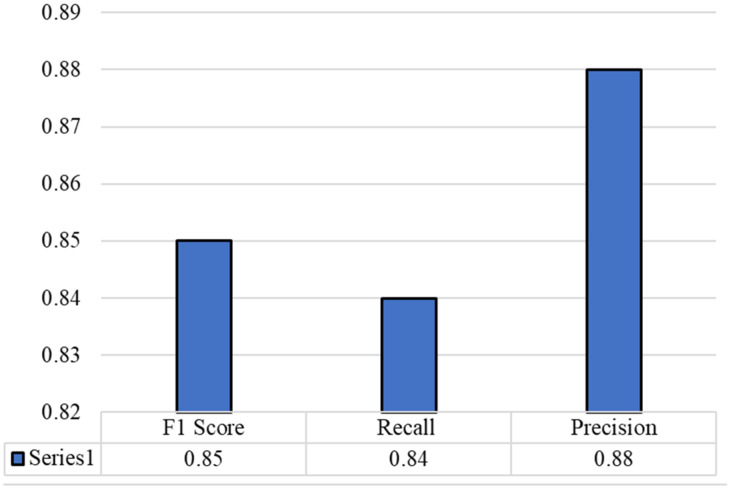
Recall, F1 score, and precision of the proposed model.

**Figure 6:**
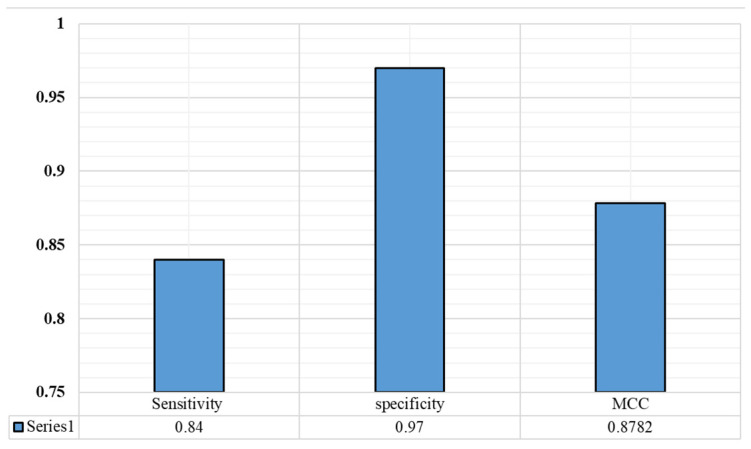
The proposed model’s sensitivity, specificity, and Matthews correlation coefficient.

We describe the architecture of the COVID-19 variant classification network in Section 3.3. In Table 1, we present a comparison between COVID-19 and different DNA/RNA analysis methods, and the statistical results support this comparison. To the best of our knowledge, there is no proposed method for the classification of variants of COVID-19 that has been developed so far. Thus, we implemented current genomic sequence analysis methods with hyperparameter tuning for the proposed task, before comparing them with the proposed method. We evaluated the proposed models with various COVID-19 variants that were collected from NCBI. The confusion matrix, recall, F1 score, and precision of the proposed model are presented in Figure 4 and Figure 5. The sensitivity, specificity, and MCC are shown in Figure 6. The results obtained in this study were compared to results achieved using nucleotide sequence data and handcrafted-feature-based and deep-learning-based methods. A composite nucleotide approach was used by [25] to predict splicing sites by comparing the nucleotide compositions of dinucleotides, trinucleotides, tetranucleotides, and their respective features. The results obtained with the optimized algorithm were tuned for COVID-19 variant classification, in which we achieved 66.9% overall accuracy for DNC, TNC, TetraNC, and composite NC, respectively. Several networks have been developed, and they can be used to predict the sequence specificities of DNA-binding proteins, such as DeeperBind [26]. For sequence representation, DeeperBind utilizes both CNN and LSTM, while DeepBind only uses CNN features. It was found that the two techniques delivered 85.6% and 89.2% accuracy for the presented task.

### 4.5. Comparison with State of the Art

The proposed technique was compared with recent SOTA techniques, and detailed ablation studies were also performed on various models, such as CNN-LSTM, CNN-GRU, CNN-BDLSTM, and CNN-BDGRU. The proposed model outperformed these models by achieving a high accuracy score of 88.36% compared with SOTA techniques.

## 5. Conclusions

COVID-19 has been successfully investigated using machine-learning-based methods using several different types of data, such as X-ray images, cough sounds, and genome sequences, which has achieved significant success; however, there is currently no method that can analyze its new variants efficiently. Therefore, we proposed a unified framework for the classification of COVID-19 variants using a temporal convolutional neural network by utilizing genome sequence data. Furthermore, we collected the most recent data for all COVID-19 variants and performed different baseline techniques to classify these variants, with our proposed classification network having the best performance. To better understand how neural networks may learn mutations and categorize a given set of sequences according to these mutations, we will attempt to include explainable artificial intelligence in COVID-19 analyses in a following study.

## Figures and Tables

**Table 1:** Details of ablation study and comparative analysis of the proposed framework with existing techniques. (*) represents the implementation of the methods is done by our team.

Method	Backbone	Overall Accuracy (%)
DNC * + SVM [34]	Statistical patterns	52.0
TNC * + SVM [34]	Statistical patterns	54.3
TetraNC * + SVM [34]	Statistical patterns	52.2
CompositeNC * + SVM [34]	Statistical patterns	57.3
DeepBind [26]	Deep model	61.3
DeeperBind [26]	Deep model	67.8
Attention-CNN-LSTM	Deep model	79.5
**Our CNN-LSTM**	Deep model	71.4
**Our CNN-GRU**	Deep model	71.1
**Our CNN-BDLSTM**	Deep model	74.8
**Our CNN-BDGRU**	Deep model	75.3
**Our proposed TCN**	Deep model	**88.36**

## Data Availability

Not applicable.

## References

[1] Chan J.F.-W., Yuan S., Kok K.-H., To K.K.-W., Chu H., Yang J., Xing F., Liu J., Yip C.C.-Y., Poon R.W.-S. (2020). A familial cluster of pneumonia associated with the 2019 novel coronavirus indicating person-to-person transmission: A study of a family cluster. Lancet.

[2] Lu R., Zhao X., Li J., Niu P., Yang B., Wu H., Wang W., Song H., Huang B., Zhu N. (2020). Genomic characterisation and epidemiology of 2019 novel coronavirus: Implications for virus origins and receptor binding. Lancet.

[3] Paraskevis D., Kostaki E.G., Magiorkinis G., Panayiotakopoulos G., Sourvinos G., Tsiodras S. (2020). Full-genome evolutionary analysis of the novel corona virus (2019-nCoV) rejects the hypothesis of emergence as a result of a recent recombination event. Infect. Genet. Evol..

[4] Wan Y., Shang J., Graham R., Baric R.S., Li F. (2020). Receptor recognition by the novel coronavirus from Wuhan: An analysis based on decade-long structural studies of SARS coronavirus. J. Virol..

[5] Letko M., Marzi A., Munster V. (2020). Functional assessment of cell entry and receptor usage for SARS-CoV-2 and other lineage B betacoronaviruses. Nat. Microbiol..

[6] Liu X., Wang X.-J. (2020). Potential inhibitors against 2019-nCoV coronavirus M protease from clinically approved medicines. J. Genet. Genom..

[7] Ozturk T., Talo M., Yildirim E.A., Baloglu U.B., Yildirim O., Acharya U.R. (2020). Automated detection of COVID-19 cases using deep neural networks with X-ray images. Comput. Biol. Med..

[8] Yan Q., Weeks D.E., Xin H., Swaroop A., Chew E.Y., Huang H., Ding Y., Chen W. (2020). Deep-learning-based prediction of late age-related macular degeneration progression. Nat. Mach. Intell..

[9] Koohi-Moghadam M., Wang H., Wang Y., Yang X., Li H., Wang J., Sun H. (2019). Predicting disease-associated mutation of metal-binding sites in proteins using a deep learning approach. Nat. Mach. Intell..

[10] Altschul S.F., Gish W., Miller W., Myers E.W., Lipman D.J. (1990). Basic local alignment search tool. J. Mol. Biol..

[11] Li M., Du X., Villaruz A.E., Diep B.A., Wang D., Song Y., Tian Y., Hu J., Yu F., Lu Y. (2012). MRSA epidemic linked to a quickly spreading colonization and virulence determinant. Nat. Med..

[12] Roux S., Adriaenssens E.M., Dutilh B.E., Koonin E.V., Kropinski A.M., Krupovic M., Kuhn J.H., Lavigne R., Brister J.R., Varsani A. (2019). Minimum information about an uncultivated virus genome (MIUViG). Nat. Biotechnol..

[13] Zielezinski A., Vinga S., Almeida J., Karlowski W.M. (2017). Alignment-free sequence comparison: Benefits, applications, and tools. Genome Biol..

[14] Alimadadi A., Aryal S., Manandhar I., Munroe P.B., Joe B., Cheng X. (2020). Artificial Intelligence and Machine Learning to Fight COVID-19.

[15] Randhawa G.S., Hill K.A., Kari L. (2020). MLDSP-GUI: An alignment-free standalone tool with an interactive graphical user interface for DNA sequence comparison and analysis. Bioinformatics.

[16] Zeng H., Edwards M.D., Liu G., Gifford D.K. (2016). Convolutional neural network architectures for predicting DNA-protein binding. Bioinformatics.

[17] Zou J., Huss M., Abid A., Mohammadi P., Torkamani A., Telenti A. (2019). A primer on deep learning in genomics. Nat. Genet..

[18] Phan D., Ngoc G.N., Lumbanraja F.R., Faisal M.R., Abipihi B., Purnama B., Delimiyanti M.K., Kubo M., Satou K. (2017). Combined use of k-mer numerical features and position-specific categorical features in fixed-length DNA sequence classification. J. Biomed. Sci. Eng..

[19] Zhang X., Beinke B., Kindhi B.A., Wiering M. (2020). Comparing machine learning algorithms with or without feature extraction for DNA classification. arXiv.

[20] Do D.T., Le N.Q.K. (2020). Using extreme gradient boosting to identify origin of replication in Saccharomyces cerevisiae via hybrid features. Genomics.

[21] Xu H., Jia P., Zhao Z. (2021). Deep4mC: Systematic assessment and computational prediction for DNA N4-methylcytosine sites by deep learning. Brief. Bioinform..

[22] Remita A.M., Diallo A.B. Statistical linear models in virus genomic alignment-free classification: Application to hepatitis C viruses. Proceedings of the 2019 IEEE International Conference on Bioinformatics and Biomedicine (BIBM).

[23] Lopez-Rincon A., Tonda A., Mendoza-Maldonado L., Mulders D.G., Molenkamp R., Perez-Romero C.A., Claassen E., Garssen J., Kraneveld A.D. (2021). Classification and specific primer design for accurate detection of SARS-CoV-2 using deep learning. Sci. Rep..

[24] Ullah W., Hussain T., Khan Z.A., Haroon U., Baik S.W. (2022). Intelligent dual stream CNN and echo state network for anomaly detection. Knowl. -Based Syst..

[25] Ullah W., Ullah A., Hussain T., Muhammad K., Heidari A.A., Del Ser J., Baik S.W., De Albuquerque V.H.C. (2022). Artificial Intelligence of Things-assisted two-stream neural network for anomaly detection in surveillance Big Video Data. Future Gener. Comput. Syst..

[26] Feng X., Tustison N.J., Patel S.H., Meyer C.H. (2020). Brain tumor segmentation using an ensemble of 3d u-nets and overall survival prediction using radiomic features. Front. Comput. Neurosci..

[27] Khan Z.A., Ullah W., Ullah A., Rho S., Lee M.Y., Baik S.W. An Adaptive Filtering Technique for Segmentation of Tuberculosis in Microscopic Images. Proceedings of the 4th International Conference on Natural Language Processing and Information Retrieval.

[28] Van Den Oord A., Dieleman S., Zen H., Simonyan K., Vinyals O., Graves A., Kalchbrenner N., Senior A.W., Kavukcuoglu K. (2016). WaveNet: A generative model for raw audio. SSW.

[29] Wei S.-E., Ramakrishna V., Kanade T., Sheikh Y. Convolutional pose machines. Proceedings of the IEEE conference on Computer Vision and Pattern Recognition.

[30] Newell A., Yang K., Deng J. (2016). Stacked hourglass networks for human pose estimation. Proceedings of the European Conference on Computer Vision.

[31] Dantone M., Gall J., Leistner C., Van Gool L. (2014). Body parts dependent joint regressors for human pose estimation in still images. IEEE Trans. Pattern Anal. Mach. Intell..

[32] Lin T.-Y., Dollár P., Girshick R., He K., Hariharan B., Belongie S. Feature pyramid networks for object detection. Proceedings of the IEEE conference on computer vision and pattern recognition.

[33] Ding L., Xu C. Weakly-supervised action segmentation with iterative soft boundary assignment. Proceedings of the IEEE Conference on Computer Vision and Pattern Recognition.

[34] Ullah W., Muhammad K., Haq I.U., Ullah A., Ullah Khattak S., Sajjad M. (2021). Splicing sites prediction of human genome using machine learning techniques. Multimed. Tools Appl..

